# Supplementary material for: MetaRibo-Seq measures translation in microbiomes
Source: Nat Commun. 2020 Jun 29;11:3268. doi: 10.1038/s41467-020-17081-z (PMC7324362; doi:10.1038/s41467-020-17081-z)
Supplement: Supplementary file 10 — Supplementary Data 7 [file 41467_2020_17081_MOESM10_ESM.zip › File2/Confidence_VeryHigh_Taxonomy/34025_out.krona.html]

Javascript must be enabled to view this page.

members
magnitude
magnitudeUnassigned
count
unassigned
taxon
rank

34025\_out

8

8
superkingdom
2

8
phylum
1239

class
8
186801

186802
8
order

8
family
541000

1263
genus
8

5
species

SRS014979\_contig\_number\_35296SRS015065\_contig\_number\_contig-100\_11675.53171SRS047014\_contig\_number\_contig-100\_2998.78246SRS097920\_contig\_number\_contig-100\_4938.4939SRS104311\_contig\_number\_26224
1638786

40519

SRS058070\_contig\_number\_25871SRS146812\_contig\_number\_39110SRS148874\_contig\_number\_contig-100\_24747.24748
3
species
